# Supplementary material for: Hybrid perovskite light emitting diodes under intense electrical excitation
Source: Nat Commun. 2018 Nov 20;9:4893. doi: 10.1038/s41467-018-07383-8 (PMC6244086; doi:10.1038/s41467-018-07383-8)
Supplement: Supplementary file 1 — Supplementary Information [file 41467_2018_7383_MOESM1_ESM.docx]

# **Hybrid perovskite light emitting diodes under intense electrical excitation**

Hoyeon Kim^1^, Lianfeng Zhao^2^, Jared S. Price^1^, Alex J. Grede^1^, Kwangdong Roh^2^, Alyssa N. Brigeman^1^, Mike Lopez^1^, Barry P. Rand^2,3^ and Noel C. Giebink^1†^

*^1^Department of Electrical Engineering, The Pennsylvania State University, University Park, Pennsylvania 16802, USA*

*^2^Department of Electrical Engineering, Princeton University, Princeton, New Jersey 08544, USA*

*^3^Andlinger Center for Energy and the Environment, Princeton University, Princeton, New Jersey 08544, USA*

Supplementary Information

Supplementary Figure 1 | Pulsed external quantum efficiency (*t*_on_ = 10 μs) at a fixed current density of 0.05 A cm^-2^ as a function of increasing time between pulses (*i.e.* decreasing duty cycle) for background bias values *V*_bias_ = 0 V and *V*_bias_ = 2.25 V





Supplementary Figure 2 | Current density of 200 μm-diameter LEDs recorded as a function of time for continuous pulsed excitation with 2 μs pulses at 20 Hz repetition rate. The degradation is irreversible and appears to be associated with Joule heating, where the temperature rise during a 2 µs pulse grows rapidly as the current density increases beyond 100 A cm^-2^ according to Fig. 6d in the manuscript.





Supplementary Figure 3 | The magnitude of the second harmonic EA signal at *λ* = 730 nm increases quadratically with the amplitude of the sinusoidal voltage dither (*V*_ac_) for modulation frequencies that are well above the characteristic timescale of ion movement (*ω* = 16 kHz here). The slope begins to change as *V*_ac_ approaches 1 V since it is no longer a perturbation to the -2 V background DC bias.

**Supplementary Figure 4** **|** **a,** The electroluminescence spectra under pulsed drive (*J* equals 0.05 A cm^-2^) exhibit a red-shift with increasing background bias indicated by the legend. **b**, A similar red-shift is predicted from transfer matrix modeling of the emission spectrum as the recombination zone moves away from the TPBi interface as indicated in the inset diagram. Modeling is carried out using the method of source terms^1^ based on refractive index dispersions measured for each layer *via* spectroscopic ellipsometry.





**Supplementary Figure 5** **|** EQE-current density product of the data in Fig. 2b shown relative to the threshold of $\eta_{\mathrm{EQE}}J_{\mathrm{th}}\approx62$ A cm^-2^ estimated for a metal-clad distributed feedback laser diode operating at 160 K based on the results in Ref. [9] from the manuscript.

**Supplementary References**

1. Benisty, H., Stanley, R., & Mayer, M. Method of source terms for dipole emission modification in modes of arbitrary planar structures. *J. Opt. Soc. Am. A.* **15,** 1192–1201 (1998).
